# Exploring PHD Fingers and H3K4me0 Interactions with Molecular Dynamics Simulations and Binding Free Energy Calculations: AIRE-PHD1, a Comparative Study

**DOI:** 10.1371/journal.pone.0046902

**Published:** 2012-10-15

**Authors:** Dimitrios Spiliotopoulos, Andrea Spitaleri, Giovanna Musco

**Affiliations:** Dulbecco Telethon Institute c/o S. Raffaele Scientific Institute, Biomolecular NMR Laboratory, Center for Translational Genomics and Bioinformatics, Milano, Italy; University of Crete, Greece

## Abstract

PHD fingers represent one of the largest families of epigenetic readers capable of decoding post-translationally modified or unmodified histone H3 tails. Because of their direct involvement in human pathologies they are increasingly considered as a potential therapeutic target. Several PHD/histone-peptide structures have been determined, however relatively little information is available on their dynamics. Studies aiming to characterize the dynamic and energetic determinants driving histone peptide recognition by epigenetic readers would strongly benefit from computational studies. Herein we focus on the dynamic and energetic characterization of the PHD finger subclass specialized in the recognition of histone H3 peptides unmodified in position K4 (H3K4me0). As a case study we focused on the first PHD finger of autoimmune regulator protein (AIRE-PHD1) in complex with H3K4me0. PCA analysis of the covariance matrix of free AIRE-PHD1 highlights the presence of a “flapping” movement, which is blocked in an open conformation upon binding to H3K4me0. Moreover, binding free energy calculations obtained through Molecular Mechanics/Poisson-Boltzmann Surface Area (MM/PBSA) methodology are in good qualitative agreement with experiments and allow dissection of the energetic terms associated with native and alanine mutants of AIRE-PHD1/H3K4me0 complexes. MM/PBSA calculations have also been applied to the energetic analysis of other PHD fingers recognizing H3K4me0. In this case we observe excellent correlation between computed and experimental binding free energies. Overall calculations show that H3K4me0 recognition by PHD fingers relies on compensation of the electrostatic and polar solvation energy terms and is stabilized by non-polar interactions.

## Introduction

Histone post-translational modifications (PTMs) constitute an important regulatory platform for processes such as gene transcription and DNA damage repair [1]. Increasing evidence suggests that deregulation of histones PTMs, caused by the malfunction of factors mediating their modification, installation, removal and/or interpretation, actively contributes to the initiation and progression of human diseases [2]. The biological consequences of histone PTMs are usually mediated by evolutionarily conserved “reader/effector” modules, such as Tudor-, Bromo- and Chromo-domains, that bind to epigenetic marks in a modification- and context-specific fashion, thus promoting chromatin changes or proteins recruitment [3]–[5].

A recent addition to the list of specialized “reader” modules recognizing the modification status of histone H3 is the plant homeodomain (PHD) finger. PHD fingers are Zn^2+^ binding domains consisting of 50–80 amino acids that form a two-stranded antiparallel β-sheet followed by an α helix. The domain is present in ∼150 human proteins, many of which act as nucleosome interaction determinants playing a fundamental role in histone recognition and epigenetic mechanisms [6]–[8]. The physiological relevance of PHD modules is highlighted by the presence of mutations targeting PHD fingers in genes such as *ING, ATRX, RAG2*, and *AIRE*, which are associated with developmental diseases and neurological and immunological disorders as well as with cancer [9]. Recent structural and functional studies suggest that the PHD finger family can be divided into several subfamilies based on their specificity towards post-translational histone modifications including the methylation status of histone lysines, such as histone H3 lysine 4 (H3K4me0 *vs* H3K4me2/3), H3 lysine 9 (H3K9me3) or H3 lysine 36 (H3K36), and to a smaller degree the methylation state of H3 arginine 2 (H3R2me0 *vs* H3R2me2) and the acetylation state of lysine K14 (H3K14) [7], [8], [10]. The best structurally characterized subfamily comprises PHD modules capable of coordinating H3K4me3/me2 through conserved aromatic side chains via π-cation interactions, like BPTF [11] and the ING PHD fingers [12], [13]. A distinct subfamily comprises the PHD module of BHC80 [14], the first PHD finger of AIRE (AIRE-PHD1) [15], [16], the first and the second PHD fingers of CHD4 [17], TRIM24 [18] and the first PHD finger of BRPF2 (BRPF2-PHD1) [19], which recognize the histone H3 tail bearing unmodified lysine 4 (H3K4me0). In this context, we and others [20], [21] showed that the first PHD finger of autoimmune regulator protein AIRE (AIRE-PHD1) recognizes H3K4me0, thus promoting the expression of its target genes. AIRE is a transcriptional activator mainly expressed in medullary thymic epithelial cells (mTEC), where it controls the expression of tissue specific antigens, thus enlarging the repertoire of antigens available for the induction of immunological tolerance, thereby preventing autoimmunity [22]. Recent studies have demonstrated that AIRE binding to hypomethylated H3 through its PHD finger module is necessary for AIRE-mediated regulation of gene expression and central tolerance induction [23]. Importantly, mutations in the *AIRE* gene [24] cause autoimmune polyendocrinopathy-candidiasis-ectodermal dystrophy (APECED) [25], [26]. At variance with what was observed for the BPTF and ING subfamily, AIRE-PHD1 does not present the typical conserved aromatic side chains used to coordinate the tri- or di-methyl ammonium ion of H3K4me3 via π-cation interactions. In AIRE-PHD1 the key elements of the methylated lysine-binding aromatic cage are substituted by negatively charged residues which can favourably interact with unmethylated H3K4me0, providing an alternative to the recognition of H3 via aromatic caging [15], [16], [20], [21]. Similarly to what has been observed for other PHD fingers in complex with methylated or unmethylated histone tails, H3K4me0 fits snugly into the PHD binding pocket forming an additional β-strand onto the existing antiparallel β-sheet of the domain (Figure S1). In order to get further insights into the molecular details at the basis of H3K4me0 read-out by PHD fingers, it is of primary importance to investigate the dynamic events characterizing histone recognition and to define the energetic parameters driving complex formation. To this end we applied multicopy molecular dynamics (MD) simulations on free and bound AIRE-PHD1 to explore the dynamic events at the basis of the AIRE-PHD1/H3K4me0 interaction. We identified the residues most affected by peptide binding in terms of conformational dynamics and investigated the perturbation of the domain slow collective motions upon peptide association. To define the dominant factors underlying H3K4me0 histone recognition, we next analysed the native and mutant complexes using Molecular Mechanics/Poisson-Boltzmann Surface Area (MM/PBSA) calculations. The MM/PBSA method is a versatile tool to calculate the binding free energies of a given protein–ligand complex; the method incorporates the effects of thermal averaging with a force field/continuum solvent model to post-process a series of representative snapshots from MD trajectories. MM/PBSA has been successfully applied to compute the binding free energy of numerous protein–ligand interactions [27]–[30]. Calculations show that H3K4me0 recognition by AIRE-PHD1 relies on compensation of the electrostatic and polar solvation energy terms and is mainly stabilized by non polar interactions. Importantly MM/PBSA calculations extended to other PHD/H3K4me0 complexes display a similar distribution in the energy contributions.

## Results

### Binding of H3K4me0 peptide changes AIRE-PHD1 dynamics

**Figure 1:**
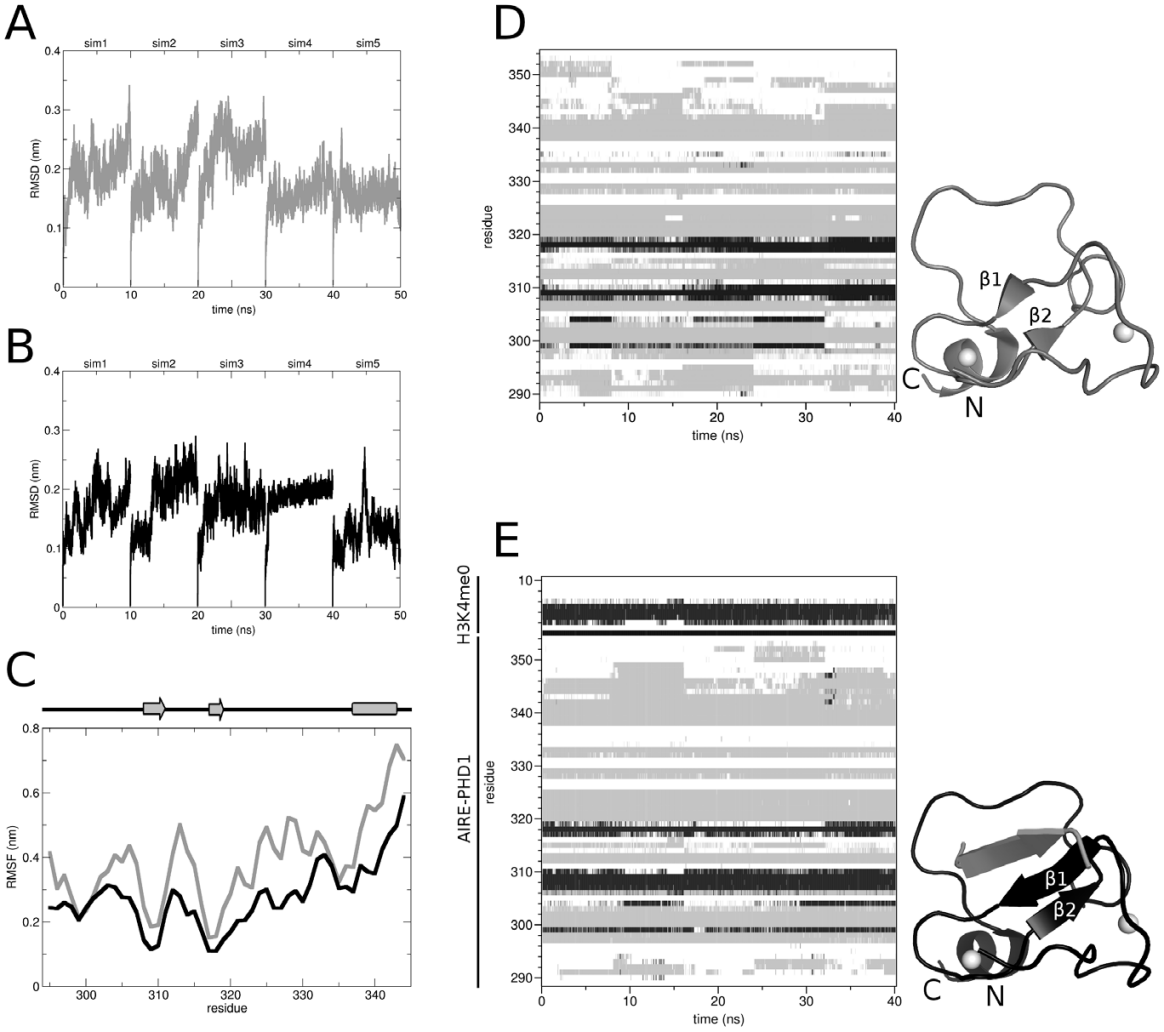
Conformational analysis of free and bound AIRE-PHD1. Cα RMSD from (A) free and (B) bound starting AIRE-PHD1 structure, as a function of time. (C) RMSF of Cα atoms from their time-averaged positions for free (grey) and bound (black) AIRE-PHD1. Secondary structure assignment for free (D) and bound (E) AIRE-PHD1 as defined by *do_dssp* [54] as a function of time: black, white and grey denote “β-sheet”, “coil”, and other secondary structure elements, respectively. For this analysis the last 8 ns of each of the five MD simulations were concatenated into a single 40-ns trajectory. Binding of H3K4me0 to AIRE-PHD1 induces the extension of the β1 strand up to residue Glu307. Two representative structures of free (grey) and bound (black) AIRE-PHD1 are shown: white spheres denote Zn^2+^ ions.

We used MD simulations to investigate possible AIRE-PHD1 responses to peptide binding in terms of structural and/or dynamic variations. In order to partially compensate for the incomplete MD sampling of individual trajectories we adopted a multi-copy approach to allow for a more exhaustive conformational sampling [31]. For each system (i.e. free and bound AIRE-PHD1) we performed five molecular dynamics simulations (10 ns each), whereby the last 8 ns of each simulation were concatenated into a single trajectory and subjected to analysis. The Cα root-mean square (RMSD) deviations from the initial structure of AIRE-PHD1 in its free (Figure 1A) and bound form (Figure 1B) appeared relatively stable with a RMSD<2.5 Å. Furthermore, the additional β strand formed by H3K4me0 onto the existing antiparallel β-sheet of AIRE-PHD1 was maintained throughout the simulation and induced extension of the β1 strand up to residue Glu307, as assessed by the secondary structure assignment along the simulation (Figure 1D,E) (we use single-letter code and three-letter code to identify peptide and protein residues, respectively).

Overall peptide binding restricted the conformational space explored by AIRE-PHD1 as assessed by a general reduction of the Cα root mean square fluctuations (RMSF) (Figure 1C, Figure S2). In particular, intermolecular interactions (summarized in Table S1) between the first 9 histone H3 residues and the binding groove (residues Asn295-Cys310, Ser332-Trp335) lowered AIRE-PHD1 flexibility in this region. Interestingly, domain regions not in direct contact with the peptide, like for example the loop connecting the two β-strands (Gly313–Arg316), also reduced their fluctuation in the complex.

**Figure 2:**
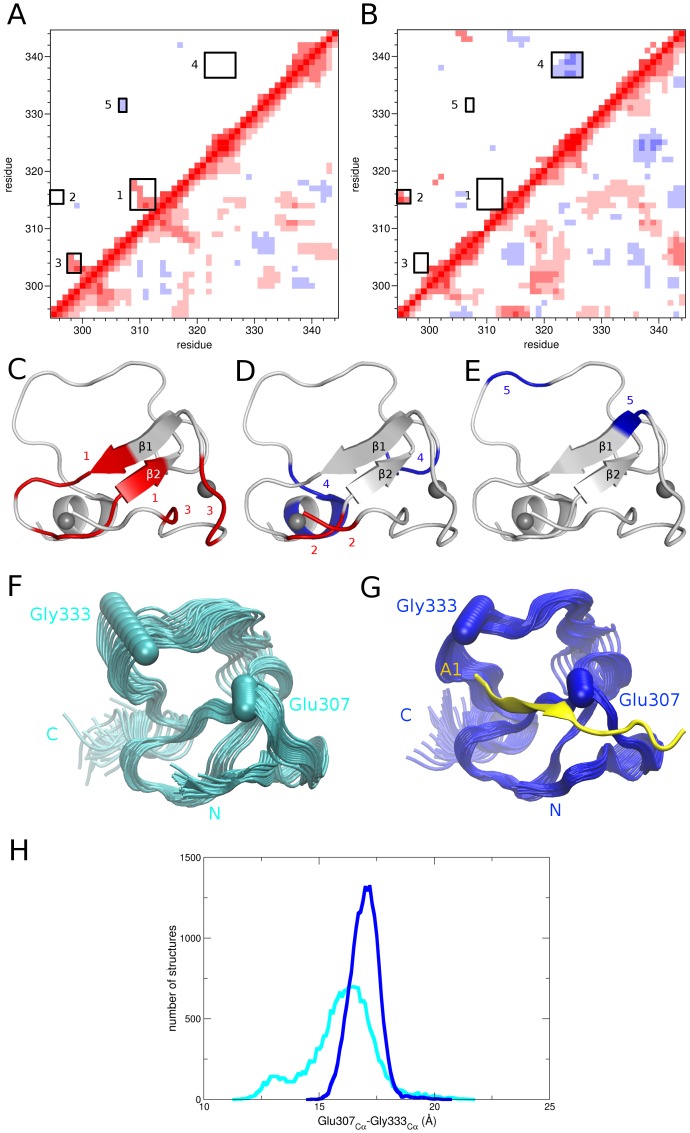
Correlated motions and PCA of free and bound AIRE-PHD1. Residue based (C_α_ atoms) correlation maps of (A) free and (B) bound AIRE-PHD1. Correlated (positive) and anticorrelated (negative) motions between atom pairs are represented as color gradients of red and blue, respectively. Above the matrix diagonal only the |Corr_ij_|>0.5 are reported. Relevant correlations/anticorrelations discussed in the text are highlighted by numbered boxes and (C–E) reported in red/blue on the AIRE-PHD1 structure. PCA analysis of free (F) and bound (G) AIRE-PHD1. Superimposition of 20 filtered configurations obtained by projecting the Cα motion of free and bound AIRE-PHD1 onto the first 6 and 4 eigenvectors, respectively. The first 6 and 4 eigenvectors obtained from the simulation of free and bound AIRE-PHD1, respectively, capture 70% of the cumulative proportion of the total variance. The Cα atoms of Glu307 and Gly333 are shown as spheres, the H3K4me0 is represented in yellow. (H) Distribution of the distances between the Cα atoms of Glu307 and Gly333 along the dynamics of free (cyan) and bound (blue) AIRE-PHD1.

We next wondered if binding of H3K4me0 peptide to AIRE-PHD1 would influence AIRE-PHD1 concerted motion. To this aim we calculated the correlation matrix Corr_ij_ for all pairs of Cα atoms. This matrix describes linear correlations between all pairs of Cα atoms as they move around their average position during the dynamics and provides a comprehensive picture of the correlated and anticorrelated motions. Comparison between the correlation matrix of free (Figure 2A) and bound AIRE-PHD1 (Figure 2B) unveiled differences in the correlated motion networks of the two systems. On the one hand the free domain showed few anticorrelated motions (Figure 2A), on the other hand, the presence of the peptide dampened down some of these motions and induced in AIRE-PHD1 a series of new correlated and anticorrelated movements (Figure 2B) reducing short range correlations and increasing concerted motions in distal residues. In detail we observed that, upon binding, the correlation between AIRE-PHD1 β-strands (box 1 in Figure 2A,C) is reduced, in favour of a new correlation between β1 and the histone H3 peptide (Figure S3). Moreover binding of the peptide induced the formation of correlated motions between Glu296 and Pro315 (box 2 in Figure 2B, D) which are linked by stable backbone interactions in the complex (Figure S4A). Conversely, salt-bridge formation between H3K9 and Glu298 reduced the correlation between Glu298-Ala300 and Arg303-Glu305, which were linked by an electrostatic interaction between Arg303 and Glu298 in the free domain (box 3 in Figure 2A, C, Figure S4B). Finally in the presence of the ligand we also observed an extensive anticorrelation between residues Cys322-Pro325 and Cys337-Cys340 (box 4 in Figure 2B, D) which is caused by the rapid formation and disruption of two hydrogen bonds between Ser324 and Ser338 (Figure S4C).

Taken together, RMSF and correlation matrix analysis reveal that binding of H3K4me0 reduces AIRE-PHD1 flexibility and increases the domain's concerted motions, both correlated and anticorrelated.

### Principal component analysis

In order to characterize the overall domain motions, we carried out Principal Component Analysis (PCA) of the covariance matrix resulting from the trajectories [32]. PCA can transform the original space of correlated variables into a reduced space of independent variables (i.e. principal components or eigenvectors). PCA identifies relevant low-energy displacements of groups of residues and emphasizes the amplitude and direction of dominant protein motions by projecting the trajectories onto a reduced dimensionality space, thus distilling the slow modes captured in the trajectories [31]. Using this approach, we have identified the protein regions involved in the most relevant collective conformational changes and shed light on the AIRE-PHD1 dynamic modulation induced by ligand binding. First of all we observed that the cumulative variance captured by the first few eigenvectors of free AIRE-PHD1 is consistently lower as compared to the AIRE-PHD1/H3K4me0 complex. In particular, in free and bound AIRE-PHD1 70% of the cumulative proportion of the total variance is captured by the first six and four eigenvectors (Table S2). This is in line with the fact that peptide binding increased AIRE-PHD1 correlated motions (Figure 2B). Consistently, comparison between simulations in terms of principal components through the calculation of their root-mean square inner product (RMSIP) [33] reveals that the essential motions characterizing free and bound AIRE-PHD1 are different (RMSIP = 0.52) and describe different essential subspaces. Projection of the MD trajectories along these components showed that the essential motion of free AIRE-PHD1 was dominated by a movement of the loop comprising residues Arg328-Thr334 towards the β1 strand (box 5 in Figure 2B,E). Notably this intrinsic domain “breathing” (Figure 2F) was strongly reduced in the presence of H3K4me0 peptide which blocked the domain in an “open” conformation (Figure 2G). Consistently we observed along the whole simulation a narrowing of the distribution of the sampled distance between the Cα atoms of Glu307 and Gly333 (Figure 2H).

### MM/PBSA calculations on native and mutant AIRE-PHD1/H3K4me0 complexes

We next investigated the energetic parameters driving the interaction between AIRE-PHD1 and histone H3 peptide both in the native and mutant forms and compared the computational results with the available experimental data. For this purpose we exploited the wealth of conformational data generated by MD simulations to perform binding free energy calculations on the wild-type complex using the MM/PBSA approach. The method expresses the free energy of binding (ΔG_comp_) as the difference between the free energy of the complex and the free energy of the receptor plus the ligand averaged over a number of trajectory snapshots [34]. Next, we performed alanine mutations on the same set of snapshots and recomputed the associated binding free energy differences (ΔΔG_comp_). Results were then compared with available experimental thermodynamic data as measured by ITC [15], [20]. The calculations were performed using in-house scripts (see Materials and Methods) that provided rapid automation of the procedure.

**Figure 3:**
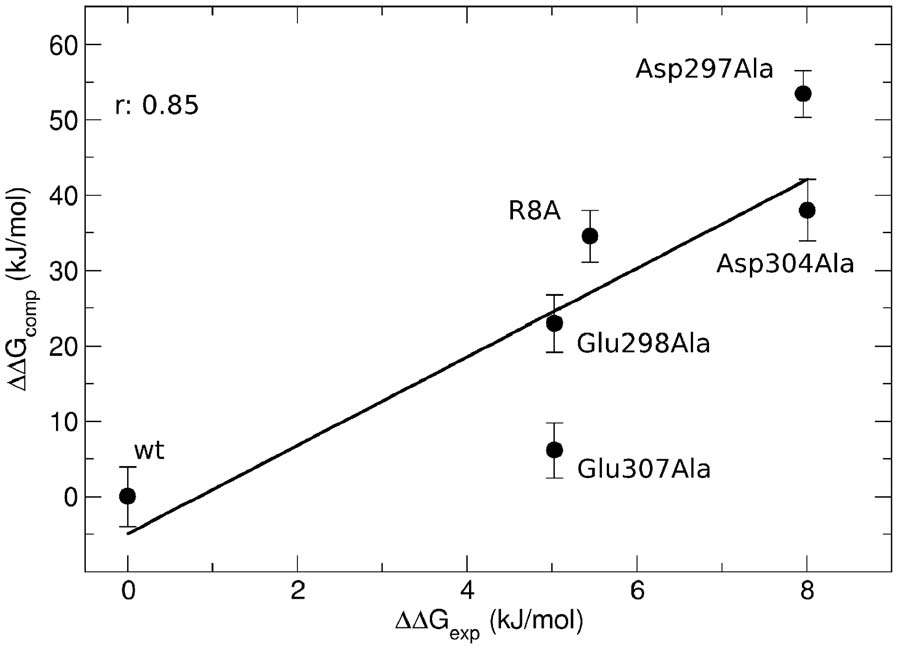
MM/PBSA calculations of native and mutant AIRE-PHD1/H3K4me0 complexes. Plot of the experimental binding free energy differences (ΔΔG_exp_) versus the calculated binding free energy differences (ΔΔG_comp_) of AIRE-PHD1/H3K4me0 alanine mutants.

It is important to note that the MM/PBSA approach allows for a rapid estimation of the variation in the free energy of binding, however it generally does not replicate the absolute binding free energy values. Nevertheless it usually exhibits good correlation with experiments, thus representing a fair compromise between efficiency and efficacy for the calculation and comparison of binding free energy variations. Indeed in our calculations we observed that the computed binding free energies were larger than those obtained by experiment (Table 1), an overestimation which has been already observed in other systems. This behaviour has been often ascribed to the omission of the entropic contribution, which is a typical approximation in these calculations [27], [35]–[39]. Herein we were mainly interested in the difference between wild-type and mutant complexes and despite the intrinsic limitations of the method, we observed good qualitative agreement between computation and experiment with a correlation coefficient *r* of 0.85 (Figure 3).

**Table 1:** MM/PBSA binding free energies (kJ/mol) of wild-type and mutant AIRE-PHD1/H3K4me0 complexes.

	mutants		Experiment		Computation		Polar contributions		Non-polar contributions	
	1ΔG_exp_	2ΔΔG_exp_	3ΔG_comp_	4ΔΔG_comp_	5ΔG_coul_	6ΔG_ps_	7ΔG_polar_	8ΔG_vdW_	9ΔG_nps_	10ΔG_nonpolar_
**wild-type**	−29.73	0	−170.7(4.0)	0	−4337.7(16.3)	4416.4(15.6)	78.7	−217.6(2.7)	−31.8(0.2)	−249.4
**Glu307Ala**	−24.70	5.0	−164.5(3.6)	6.1	−3779.3(16.4)	3860.0(16.5)	80.6	−214.2(2.8)	−30.9(0.2)	−245.1
**Glu298Ala**	−24.70	5.0	−147.7(3.8)	22.9	−3632.3(16.4)	3742.5(15.1)	110.2	−226.6(2.6)	−31.3(0.2)	−257.9
**R8A**	−24.28	5.5	−136.1(3.4)	34.5	−3384.0(13.4)	3486.6(12.7)	102.6	−209.3(2.5)	−29.5(0.1)	−238.7
**Asp304Ala**	−21.77	8.0	−117.2(3.1)	53.4	−3681.2(16.8)	3817.2(16.0)	136	−222.1(2.6)	−31.2(0.2)	−253.2
**Asp297Ala**	−21.72	8.0	−132.7(4.1)	38	−3507.5(16.0)	3634.3(15.2)	126.8	−228.0(2.5)	−31.5(0.2)	−259.5

1 experimental binding free energy as measured in [15], [20].

2 difference between wild-type and mutant experimental binding free energies.

3 computational binding free energies.

4 difference between computational binding free energies of wild-type and mutant complex.

5 coulombic term.

6 polar solvation term.

7 polar term (sum of coulombic and polar solvation terms).

8 van der Waals term.

9 non-polar solvation term.

10 non-polar term (sum of van der Waals and non polar solvation terms).

Standard errors are given in parentheses.

Breakdown of the binding free energy into its components, including van der Waals, electrostatic, polar solvation, and non-polar solvation interaction energy terms, identified the factors dominating binding affinity for both wild-type and mutants. On the one hand, coulombic (ΔG_coul_), van der Waals (ΔG_vdW_) and non-polar solvation terms (ΔG_nps_) favoured complex formation. On the other hand, binding was strongly antagonized by positive polar solvation contributions (ΔG_ps_), due to the unfavourable desolvation energy of the polar and charged residues of both the peptide and AIRE-PHD1. Overall this high desolvation energy penalty could not be completely compensated by the coulombic interactions, thus leading to an unfavourable contribution of the polar terms to the binding free energy change (ΔG_polar_>0). Conversely, the non polar energy terms ΔG_nonpolar_, formed by the sum of van der Waals and non-polar solvation terms (which is much smaller than the van der Waals term) promoted complex formation (ΔG_nonpolar_<0). Taken together, these data indicate that the complex is stabilized by non-polar interactions and modulated by polar contributions (Table 1). Overall alanine mutations decreased both the coulombic and polar solvation contributions with respect to the wild-type complex, resulting in an overall increase of the positive polar terms, thus antagonizing binding. Consistently, ΔG_polar_ correlated well with experimental results (*r* = 0.83), whereas ΔG_nonpolar_ did not show substantial variation.

### MM/PBSA calculations on other PHD fingers recognizing H3K4me0

**Figure 4:**
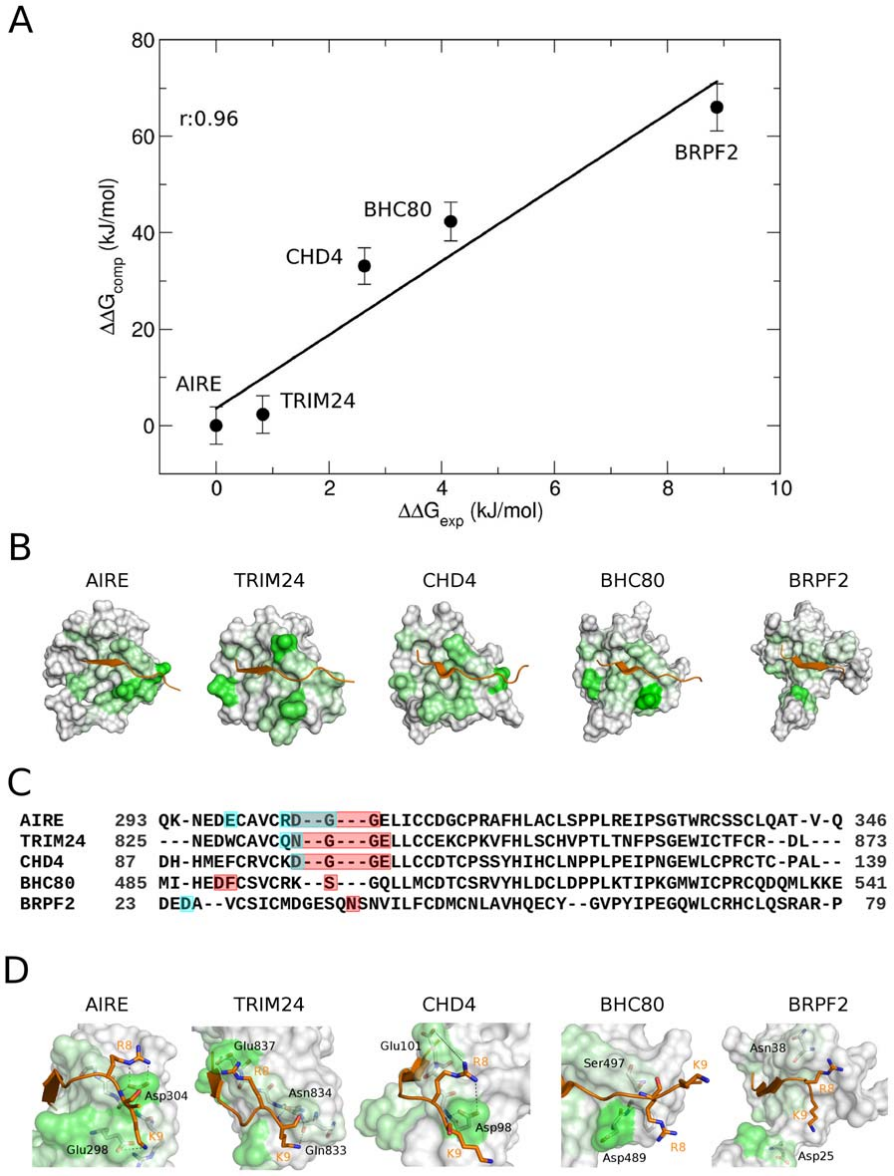
MM/PBSA calculations of PHD fingers recognizing H3K4me0. (A) Correlation between the experimental binding free energy (ΔG_exp_) and the calculated binding free energy (ΔΔG_comp_) of H3K4me0-binding PHD fingers. (B) Representation of the energetic contributions (coulombic and van der Waals energies) associated with peptide-domain intermolecular contacts. For clarity, normalized interaction energies are mapped only on the PHD finger surface in a range from white (no contribution) to green (high contribution). H3K4me0 is represented as orange ribbon. (C) Structural alignment of the H3K4me0-binding PHD fingers generated by MultiSeq [55]. Residues interacting with H3R8 and H3K9 are highlighted in red and cyan, respectively; residues interacting with both H3R8 and H3K9 are highlighted in grey. (D) Representation of intermolecular interactions between H3R8 and H3K9 (shown in sticks) and PHD finger residues; dashed lines indicate polar contacts.

Prompted by the encouraging results obtained on AIRE-PHD1/H3K4me0 complexes, we next wondered if the MM/PBSA method could be also applied to calculate and rank the ΔG of binding of other PHD fingers in complex with H3K4me0 peptide, including CHD4-PHD2, BHC80-PHD, TRIM24-PHD and BRPF2-PHD1 (Table 2). The last nanosecond of the five 10 ns-long MD simulations was concatenated into a single trajectory (5 ns), which was used for MM/PBSA calculations. Despite the fact that different experimental conditions (where the main difference regarded the type of buffer and the ionic strength, whereas as both pH and temperatures were comparable) and techniques (fluorescence and ITC) were used to determine the dissociation constants (Table 2 and Table S3), we observed a good correlation between experimental and computational ΔΔG of binding (*r* = 0.96) (Figure 4A). Importantly, in all the five complexes recognition of H3K4me0 was stabilized by the non-polar term (ΔG_nonpolar_<0), whereas the ΔG_polar_ term consisting of negative electrostatic and positive polar solvation components antagonized binding. Notably, ΔG_polar_ correlated well with the ΔΔG_exp_ (*r* = 0.88), whereas ΔG_nonpolar_ did not change significantly among the different complexes (Table 2).

**Table 2:** MM/PBSA binding free energies (kJ/mol) for PHD finger/H3K4me0 complexes.

		Complex with H3K4me0		Experiment		Computation		Polar contributions		Non-polar contributions	
	1ΔG_exp_	2ΔΔG_exp_	Techniques and Experimental Conditions	3ΔG_comp_	4ΔΔG_comp_	5ΔG_coul_	6ΔG_ps_	7ΔG_polar_	8ΔG_vdW_	9ΔG_nps_	10ΔG_non polar_
**AIRE**	−29.73	0	ITC, 20 mM phosphate buffer, 150 mM NaCl, 2 mM 2-mercaptoethanol, 50 mM ZnCl_2_ pH 7.2	−170.1(3.9)	0	−4337.7(16.3)	4417.0(15.7)	79.3	−217.6(2.7)	−31.8(0.2)	−249.4
**TRIM24**	−28.90	0.83	ITC, 20 mM phosphate buffer, 150 mM NaCl, 2 mM 2-mercaptoethanol, 50 mM ZnCl_2_ pH 7.2	−167.8(3.9)	2.3	−4506.5(20.9)	4562.8(18.4)	56.3	−195.0(2.4)	−29.2(0.1)	−224.2
**CHD4**	−27.10	2.63	Tryptophane fluorescence, 20 mM sodium phosphate, 150 mM NaCl, 10 mM DTT, 1 mM NaN3, pH 7.2	−137.0(3.8)	33.1	−4035.3(27.6)	4156.0(23.7)	120.7	−226.2(3.7)	−31.5(0.1)	−257.6
**BHC80**	−25.57	4.16	ITC, 25 mM Tris-HCl, 50 mM NaCl, 2 mM 2-mercaptoethanol, pH 7.2	−127.8(4.0)	42.3	−2245.8(11.0)	2355.5(10.6)	109.7	−208.8(3.9)	−28.7(0.1)	−237.5
**BRPF2**	−20.85	8.88	ITC, 50 mM Tris-HCl, 100 mM NaCl, pH 7.5	−104.1(4.9)	66	−4582.6(34.7)	4732.8(32.4)	150.2	−221.0(3.7)	−33.4(0.2)	−254.3

1 experimental binding free energy.

2 difference between AIRE-PHD1 and PHD finger complex experimental binding free energies.

3 computational binding free energies.

4 difference between the computational binding free energy of AIRE-PHD1/H3K4me0 and other PHD/H3K4me0 complex.

5 coulombic term.

6 polar solvation term.

7 polar term (sum of coulombic and polar solvation terms).

8 van der Waals term.

9 non-polar solvations term.

10 non-polar term (sum of van der Waals and non polar solvation terms).

Standard errors are given in parentheses.

### Analysis of the intermolecular contacts

Taking advantage of the MD simulations performed on the five different complexes, we performed a comparative analysis of the energetic contributions (coulombic and van der Waals energies) associated with peptide-domain intermolecular contacts. These contacts were defined on the basis of their intermolecular distances (<3 Å) and their statistical occurrence (>30%) during the MD simulations, and their associated energies (Table S1) were mapped onto the domain surface (Figure 4B). Interestingly, we observed that overall the first 6 residues of the histone peptide established similar contacts with equivalent residues, as defined by a structure-based alignment (Figure 4C). Remarkably, the main differences involved residues H3R8 and H3K9 (H3A7 had negligible contributions), which displayed a higher number of contacts (Table S1) and favourable interaction energies (Figure 4B,D; Table S1) in those PHD fingers showing the best affinities towards H3K4me0. Conversely, PHD fingers lacking these interactions, like BRPF2-PHD1, displayed the lowest affinity. Overall these results suggest that recognition of H3R8 and H3K9 might regulate the fine details of the interaction, thus conferring reading specificity of different PHD domains.

## Discussion

The structure and function of PHD fingers have been under intensive investigation in the past few years resulting in more than 30 complex structures which offer a “static description” of the interaction with histone peptides [7], [10], [40]. Surprisingly, until now very few efforts have been dedicated to the dynamic characterization of these complexes and to the definition/prediction of the energetic parameters driving complex formation. In this context computational methods offer the opportunity to directly observe the binding events and to dissect the dynamic and energetic determinants dictating domain-peptide recognition. As a first step towards the dynamic and energetic understanding of histone H3 recognition by PHD fingers we first focused on dynamic and thermodynamics investigations of AIRE-PHD1 in complex with H3K4me0. AIRE-PHD1 represents a paradigmatic example of a non-modified histone H3 reader. The thermodynamic results were then compared to calculations performed on other PHD fingers belonging to the same class of H3K4me0 readers.

Multicopy MD simulations performed on AIRE-PHD1 showed that binding to H3K4me0 peptide overall diminished AIRE-PHD1 flexibility. In particular regions directly in contact with H3A1 and H3K4, including the N-terminal residues and residues located on the loop Ser332-Trp335 showed a reduction of the RMSF fluctuations (Figure 1C, Figure S1). This observation is in perfect agreement with previous ^15^N relaxation studies which showed that the very same regions became more rigid on the nanosecond time scale upon substrate recognition, as assessed by their increased heteronuclear NOE values in the presence of the peptide [15]. Analysis of the correlation maps of both free and bound AIRE-PHD1 shows that binding of H3K4me0 peptide dampens down some short-range correlations and induces new long-range correlations involving both residues in direct contact with the peptide and distal to the interaction surface, thus bridging some of the originally distant residues.

To examine the concerted motions in AIRE-PHD1 and to assess how these movements were influenced by peptide binding we used PCA. In particular, analysis of the essential domain motions highlighted the presence of a “flapping” movement in free AIRE-PHD1 involving the loop comprising residues Arg328-Thr334 and the N-terminal edge of β1. Importantly, this intrinsic domain “breathing”, which might be relevant for domain function, was blocked in an open conformation upon H3K4me0 binding. Overall, peptide recognition strongly influenced the domain network of dynamically correlated amino acids, changing the domain structure. We hypothesize that this kind of dynamic event might happen in the context of the full length AIRE and might have a functional relevance in the framework of AIRE-chromatin interaction. A challenge that remains is to determine to what degree perturbing this network may contribute to modulate domain function and/or its interactions with the tail of histone H3.

The link between dysfunction of the PHD finger activities and numerous diseases suggests a strong therapeutic potential for these systems [7]. Herein AIRE-PHD1 constitutes a useful benchmark for the generation of computational models of the interaction between PHD fingers and non methylated histone tails, as several experimental thermodynamic data on native and mutant complexes in extremely controlled condition (in terms of pH, temperature and ionic strength) are available [15], [20]. Taking advantage of both the experimental information and the wealth of conformational data obtained from our MD simulations, we adopted the MM/PBSA approach to calculate the binding free energy associated with the native and alanine mutants of AIRE-PHD1/H3K4me0 complexes. The calculated binding free energies showed good correlation with experimental data (*r* = 0.85), showing that the method was reliable for estimating binding free energy *in silico*. For both the native and mutant complexes the non-polar energy values, which were obtained from the sum of the solvent accessible surface area (SASA), and the van der Waals terms favourably contributed to the complexes' binding free energy. On the other hand, the polar energy term considerably antagonized peptide binding to both native and mutant AIRE-PHD1. This effect is mainly due to the high cost of the desolvation term of the polar residues, which is higher with respect to the favourable coulombic interaction energy, implying that intermolecular electrostatic interactions between AIRE-PHD1 and H3K4me0 peptide are insufficient to completely pay for the desolvation penalty. Nevertheless the unfavourable ΔG_polar_ term is largely compensated by the negative ΔG_nonpolar_ term resulting in a highly favourable binding free energy. Therefore complex formation is stabilized by non-polar interactions and modulated by polar ones. This thermodynamic feature has been already observed in the Tudor domain of JMJD2A in complex with H3/H4 histone tails [41] and might be a common theme in the recognition mechanism of histone tails by epigenetic readers. As expected alanine mutations diminished the electrostatic contribution to the binding free energy and decreased the polar solvation term, resulting in an overall increase of the unfavourable polar term ΔG_polar_ which antagonized binding; the non-polar contributions were mainly unaffected by the alanine mutations. Based on these results we hypothesize that in future MM/PBSA might represent a robust method to predict *in silico* the effect of other mutations and of epigenetic modifications on the ability of AIRE to decode histone H3. In particular, in the context of molecular studies focusing on the patho-physiological role of AIRE in transcriptional activation MM/PBSA calculations might constitute a rapid tool to assist experimentalists in the rational design of AIRE mutants with altered histone H3 binding activity to modulate its transcriptional activity.

Once we demonstrated that MM/PBSA could constitute a reliable computational method to calculate the binding free energy for our model system, we next wondered whether the tool could be generally applied to the energetic analysis of other PHD fingers recognizing H3K4me0. Remarkably, we observed excellent correlations between computed and experimental binding free energies (*r* = 0.96), whereby in all the PHD fingers recognition of H3K4me0 was stabilized by the non- polar term (ΔG_nonpolar_<0), whereas the ΔG_polar_ term disfavoured binding (ΔG_polar_>0). Notably ΔG_polar_ correlates well with experimental results (*r* = 0.88) suggesting that differences in the electrostatic properties of PHD fingers might influence binding affinity. To further investigate this aspect we analysed the MD simulations of the five complexes scrutinizing the interaction energies of a subset of interface residues. This analysis pointed out that the first 6 residues of H3K4me0 established similar contacts with equivalent residues in the different PHD fingers. Importantly, those complexes having higher affinity to H3K4me0 (e.g. AIRE-PHD1) displayed higher interactions with residues H3R8 and H3K9 as compared to those PHD fingers displaying lower affinity (e.g. BRPF2-PHD1). These observations suggest that H3R8 and H3K9 might be involved in the fine-tuning of PHD finger recognition, thus determining selectivity within this class of H3K4me0 readers. The observation that H3R8 alanine mutation reduces H3 affinity for AIRE-PHD1, as assessed both computationally and experimentally, supports this hypothesis. Overall these data are extremely encouraging for the application of MD combined to MM/PBSA as a valuable tool to rapidly analyse the energetic determinants dictating histone decoding in this class of epigenetic effectors.

As already demonstrated for AIRE-PHD1, it is conceivable that MM/PBSA can also be applied to this subset of histone readers to predict *in silico* the effect of alanine mutations and to characterize the fine thermodynamic details governing histone H3 recognition by single members of this subclass of epigenetic readers. PHD fingers are indeed emerging as druggable classes of protein-protein interaction domains and they represent a new frontier in drug discovery that has a huge potential for the development of future therapeutics [42]. Having established both the common principle and the differences governing histone recognition by the different members of this class of PHD fingers, this work paves the way for further investigations on other H3K4me0 readers and might assist drug design studies focusing on the development of small PHD inhibitors. It will be interesting in future to test the robustness of the method verifying whether this group of complexes can be used as a training set to predict the affinity ranking of other PHD fingers of the same class (e.g. Sp140, NSD1, Sp110). Future research will be also dedicated to explore the application of these computational methods to the energetic analysis of PHD fingers recognizing other epigenetic signatures such as the H3K4me3, H3K4me9 or acetylation mark, since several high-resolution structures of complexes are available.

## Materials and Methods

### AIRE-PHD1 MD Simulations

AIRE-PHD1 structures in free form (1xwh) and bound to H3K4me0 peptide (residues 1–10) (2ke1) were used for MD simulations. MD simulations were performed using Gromacs 4.0.7 package [43] with the optimized parameters for liquid simulation (OPLS) force field [44]. The system was neutralized by adding the appropriate number of sodium counter ions. Energy minimization procedures and a positional restraint phase of 200 ps were performed to relieve unfavourable interactions; a MD simulation of 300 ps was subsequently performed in the NVT ensemble to equilibrate the system (T = 296 K). The production runs were performed in the NPT ensemble, with P = 1 bar and T = 296 K, for 10 ns, with a time step of 2 fs. The long-range electrostatics were treated with the Particle Mesh Ewald (PME) method using 1.1 nm cut-off. For Lennard-Jones interactions a 1.1 nm cut-off was employed. The pair list was updated every 10 MD steps. Five independent 10 ns long MD simulations (50 ns production run) were performed to allow for better conformational sampling and to have a statistical validation of the binding free energies. The starting structures for the five independent simulations were extracted from the deposited NMR bundles.

Structural and energetic convergence were assessed by calculating the cumulative averages of the RMSD values from the average structure and of the MM/PBSA ΔG_bind_ values as a function of time, respectively (Fig. S5A,B).

To further verify convergence we also extended the simulation length up to 50 ns for each replica. After 50 ns all simulations had a stable RMSD profile (Figure S6). As the conformational space explored by 10 and 50 ns simulations was the same (see further) conformational analysis was performed on the 10 ns long simulations.

### MD Simulations on other PHD fingers in complex with H3K4me0 peptides

MD simulations were performed as described above on CHD4-PHD2 (2l75), BHC80-PHD (2puy), TRIM24-PHD (3o37) and BRPF2-PHD1 (2l43) in complex with H3K4me0 peptides. For CHD4-PHD2 in complex with H3 peptide, methyls were removed from H3K9 before performing MD simulations. For TRIM24-PHD only residues Asn825-Pro885 were used in the calculations, based on the structural observation that the bromodomain is not involved in PHD-H3 peptide interaction [18]. For BRPF2-PHD1, residues Gly13-Ser18 were removed to generate two separated chains comprising the PHD finger and the 12 residue-long histone tail (the structure was determined using a fusion construct, bearing both the PHD finger and the histone tail, whereas binding affinity has been determined by titrating BRPF2-PHD1 with the corresponding histone peptide [19]). Five independent 10 ns long MD simulations (50 ns production run) were performed for each complex. For NMR determined complexes five starting structures were extracted from the deposited structure ensembles (2l75, 2l43), whereas for the crystallographic structures (2puy, 3o37) different seed numbers were used for five independent dynamics runs. Structural and energetic convergence was assessed calculating the cumulative averages from the average structure of the RMSD and MM/PBSA ΔG_bind_ values (Fig. S5B,A).

### Principal Component Analysis

Principal component analysis (PCA) of the covariance matrix identifies dominant low-frequency, large scale motions along a trajectory generated by molecular dynamics simulations. This statistical method is used to describe the most relevant correlated motions using a new basis set directly reflecting the collective motions undergone by the system. The method allows filtration of the noise from the dominant modes determining the system motions, thus reducing the dimensionality of MD trajectories [32]. PCA requires the construction of the covariance matrix *cov* based on the 3D positional fluctuations of a given set of atoms from their ensemble average position, after removing overall rotational and translational motions by means of least-squares fitting. The elements *s_ij_* of the matrix *cov* are (equation 1):
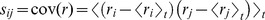
(1)where *r_i_* and *r_j_* are the vectors' Cα*_i_* and Cα*_j_* positions during the trajectory, respectively; *<r_i_>_t_* and *<r_j_>_t_* are *r_i_* and *r_j_* time averaged (*t*) over the MD trajectory; the indexes i and j denote amino acid residues. Data are typically represented by correlation maps calculated according to the correlation matrix whose elements are defined by
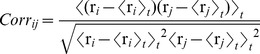
(2)


This map allows identification of pairs/groups of residues with correlated (in the same direction) and anti-correlated (in opposite directions) motions. Diagonalization of the covariance matrix c*ov* provides an orthogonal set of eigenvectors, each defined by an eigenvalue, representing the direction and amplitude of the motion, respectively, whereby the first eigenvector represents the largest contribution to the total fluctuation of the system, the second eigenvector the second largest contribution, and so on. PCA calculations were performed on the Cα coordinates of AIRE-PHD1 (residues Asn295-Thr344) structure ensembles generated by the concatenation of the last 8 ns of the five MD of free AIRE-PHD1 and in complex with H3K4me0 peptide, respectively.

#### Comparison between essential subspaces and convergence assessment

In order to compare the essential subspaces described by the eigenvectors identified in the simulations of free and bound AIRE-PHD1, we calculated the overlap between two subspaces by computing the root mean square inner product (RMSIP) between the two corresponding sets of eigenvectors:
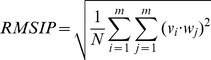
(3)where v_i_ and w_j_ are the *i*-th and *j*-th eigenvectors of the two sets. RMSIP ranges from zero (for orthogonal, non overlapping subspaces) to one (i.e., identical subspaces). The RMSIP is usually calculated on the ten eigenvectors with the largest eigenvalues defining the essential subspace [33].

Calculation of the RMSIP can also be used to compare eigenvectors describing different time windows of the same simulations in order to verify simulation convergence [45].

To assess simulation convergence we therefore divided the single simulations into three different time windows (2–5 ns; 2–8 ns; 2–10 ns) and we calculated their corresponding eigenvectors. We next calculated the RMSIP to verify whether the single subspaces described by these eigenvectors were similar. Importantly, all the RMSIP values were >0.85 indicating a large overlap between the subspaces described by these eigenvectors (Table S4). Importantly, the subspaces described by the 10 ns and 50 ns simulations are substantially the same, as indicated by the high RMSIP values between the first 10 eigenvectors of the two simulations (Table S5). We conclude that at 10 ns the simulations have reached convergence and that the 2–10 ns production runs provide adequate sampling for a meaningful analysis of these systems.

### MD-Based Binding Free Energy Calculations

The method for determining the binding free energy following the MM/PBSA approach has been described previously [34]. The binding free energy of a protein molecule to a ligand molecule in a solution is defined as:
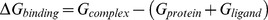
(4)


A MD simulation is performed to generate a thermodynamically weighted ensemble of structures (in our case, an ensemble of time-equidistant snapshots). The free energy term is calculated as an average over the considered structures:
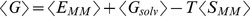
(5)


The energetic term E_MM_ is defined as:
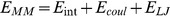
(6)where E_int_ indicates bond, angle, and torsional angle energies, and E_coul_ and E_LJ_ denote the intramolecular electrostatic and van der Waals energies, respectively.

The solvation term G_solv_ in Eq. (7) is split into polar G_polar_ and non polar contributions, G_nonpolar_ [34]:
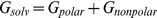
(7)


In this work G_polar_ and G_nonpolar_ were calculated with APBS (Adaptive Poisson-Boltzmann Solver program) [46]. The polar contribution G_polar_ refers to the energy required to transfer the solute from a continuum medium with a low dielectric constant (ε = 1) to a continuum medium with the dielectric constant of water (ε = 80). G_polar_ was calculated using the non linearized Poisson Boltzmann equation. The grid spacing was automatically set to an upper limit of 0.5 Å. The temperature was set to 296 K, and the salt concentration was 0.15 M. The non-polar contribution G_nonpolar_ was considered proportional to the solvent accessible surface area (SASA):
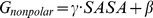
(8)where *γ* = 0.0227 kJ mol^−1^ Å^−2^ and *β* = 0 kJ mol^−1^ [47]. The dielectric boundary was defined using a probe of radius 1.4 Å.

Binding free energy calculations based on the MM/PBSA approach can be performed either according to the three trajectories method (TTM) or according to the single trajectory method (STM). The TTM requires three separate MD simulations on the three system components (the complex, the free ligand and the free receptor). This is a computationally demanding approach and prone to structural noise [28], [30]. Conversely, the STM requires a single trajectory run for the complex, whereby both the protein and ligand structures are extracted directly from the complex structure [28], thus zeroing out the E_int_ term. In this case, the protein and the ligand are assumed to behave similarly in the bound and in the free forms. This assumption is reasonable for PHD fingers, as they do not undergo structural rearrangements upon binding [6]–[8]. In the case of the peptide structural rearrangements occur upon binding to the PHD finger. In the systems under investigation the peptides always adopt a β-strand conformation when bound to the PHD finger. It is therefore reasonable to assume that the entropic term in the different complexes is very similar and will reasonably cancel out when calculating ΔΔG_comp_. The differences have been calculated using AIRE-PHD1/H3K4me0 as reference (ΔΔG_comp_ = ΔG_AIRE_−ΔG_PHD_). MM/PBSA calculations were therefore performed according to the STM protocol.

Within the MM/PBSA approximation <E_MM_> + <G_solv_> account for the enthalpy change associated with complex formation. The computational determination of the free energy of binding requires the calculation of the entropic contributions to complex formation, including conformational changes in rotational, translational and vibrational degrees of freedom of solute. Solute entropic contributions are usually estimated by either the quasi-harmonic approach (e.g., Schlitter equation) or by normal mode analysis [48]. Entropy calculations require a full sampling of the free energy landscape, an extremely computationally demanding step, which can result in unreliable results [36] with standard errors usually with an order of magnitude larger than those associated with the other energetic terms [49]. In addition, the normal mode analysis estimation is often extremely qualitative [50] and the configuration entropy estimate on a short dynamic time range can be non-significant [51]. Based on these considerations and on the fact that we were mainly interested in ΔΔG_comp_ (see further) we decided to neglect the entropic term in our calculations. The average of the last nanosecond of the five MD simulations (i.e., 125 equally time-distant frames) was considered for MM/PBSA calculations. The standard errors (SE) were calculated as follows:
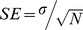
(9)where σ is the standard deviation and N is the number of structures (125) used in the calculation.

### Alanine mutations

In the absence of major conformational changes upon alanine mutation of the wild-type complex it is possible to perform binding free energy calculations of alanine mutant complexes using the MM/PBSA approach on snapshots taken from the wild-type simulation, instead of performing simulations on the single mutant complexes [29], [34]. The protocol has been successfully applied to study a variety of protein-protein interactions [28]–[30]. Briefly, the wild-type structures obtained from MD simulations are post-processed to introduce alanine mutations. Computational binding free energy values can be therefore expressed as differences in the binding free energy between wild-type and mutant complex (ΔΔG_comp_). In these calculations the entropy is neglected, assuming that the entropic contributions to the binding free energies of similar ligands cancel out upon relative comparison [28], [30], [34], [37], [52].

### GMXAPBS tool

Despite the popularity of the freely available software Gromacs 4.0.7 [43] and APBS [46] nothing free is available to automatically combine the two programs in order to directly use the MD output as input for binding free energy calculations. To facilitate the interface between the two programs, we wrote a series of Bash/Perl scripts to directly perform MM/PBSA calculations on structures generated by MD simulations. Only three MD simulation files are required to run the scripts: the trajectory file (TRR or XTC), the topology file (TPR) and the index file (NDX). For customized force fields the tool requires also the topology and parameter files. The calculations are organized in an automatic fashion that can be run in parallel in a PBS queue system. Figure S7 summarizes a schematic representation of the script work-flow. Briefly, structures generated by Gromacs MD simulations are extracted from the trajectory as PDB files. Next, the structures undergo energy minimization (the length can be determined by the user), during which the van der Waals term is calculated in double precision. The PDB files are then converted into PQR files by the *editconf* Gromacs tool. APBS performs the calculation of the solvation (polar and non-polar) and electrostatic terms by the APBS accessory program *coulomb*. The Poisson-Boltzmann equation requires generation of a box with a suitable grid. For this purpose the extreme coordinates of the protein in each dimension are automatically extracted from the structure files. 20 and 10 Å are added to each value to set the limits of the coarse and fine grids, respectively. Then, our tool automatically calculates the number of grid points that is feasible for APBS calculations with the mesh lower than 0.5 Å. Finally, when all calculations are completed, all the binding energy terms are summed up to obtain the binding free energies as the average values along the trajectory. Calculations of 3000 structures/day have been carried out in our cluster (HP 300 cores, 2.9 GHz, 4 Gb RAM, InfiniBand connectivity, Maui/TORQUE queue system). The GMXAPBS tool has been also adapted to perform alanine mutations as a post processing protocol: the GMXAPBS scripts can mutate any amino acid into alanine, truncating the residues of the protein up to the C_β_ atom and adding the missing hydrogen atom to complete the tetrahedral coordination of the C_β_ atom. Subsequently, MM/PBSA calculations are performed to calculate the energetic impact of alanine mutation on binding affinity. GMXAPBS is fully automatic and is easily adaptable to any protein-protein and protein-ligand system. The scripts are extensively commented to facilitate their customization, and the outputs are either text or pdf files reporting the calculated terms. The tool is available upon request.

### Figure preparation

All figures were created with PyMol (http://pymol.org), VMD [53]. Graphs were created using Xmgrace (http://plasma-gate.weizmann.ac.il/Grace/).

## Supporting Information

Figure S1 **Surface plot of the AIRE-PHD1/H3K4me0 complex.** Complex of AIRE-PHD1 (white cartoon and surface) and H3K4me0 (orange cartoon). AIRE-PHD1 residues interacting with H3A1, H3R2, H3K4, H3R8 and H3K9 are shown as green, magenta, cyan, pink and violet sticks, respectively. Dashed lines indicate a selection of the polar contacts of the complex, and Zn^2+^ ions are represented by grey spheres.
(TIFF)

Figure S2 **RMSF of Cα atoms from their time-averaged positions for the five replicas of free (cyan) and bound (blue) AIRE-PHD1.**
(TIFF)

Figure S3 **Residue-based (Cα atoms) correlation maps of AIRE-PHD1 (residues 295 to 344) and H3K4me0 (residues 1 to 5, black line).** An arrow indicates the correlation between AIRE-PHD1 β1 strand and the additional β strand formed by the histone tail.
(TIFF)

Figure S4 **Interactions corresponding to the correlations described by boxes 1, 2 and 3 in** **Figure 2** **.** On the left side are shown the distribution of specific distances along the dynamics of free (cyan) and bound (blue) AIRE-PHD1, on the right side are shown two representative structures for free (cyan) and bound (blue) AIRE-PHD1, with grey spheres and yellow cartoon denoting Zn^2+^ ions and histone tail, respectively. (A) Interaction between the backbone atoms of Glu296 and Pro315, (B) salt-bridge between Glu298 and Arg303 side-chains, (C) Hydrogen bonds between Ser324 and Ser338.
(TIFF)

Figure S5 (A) Cumulative averages of the RMSD values of Cα atoms relative to the average structure obtained from the simulations of the different systems. (B) Cumulative averages of the MM/PBSA values obtained for the five simulations of the different systems.
(TIFF)

Figure S6 **Cα RMSD from (A) free and (B) bound starting AIRE-PHD1 structure, as a function of time in 50 ns trajectories.**
(TIFF)

Figure S7 **Schematic diagram of GMXAPBS workflow.**
(TIFF)

Table S1 **Interatomic contacts between PHD fingers and H3K4me0 during MD simulations and associated interaction energies.** Analyses were performed on the last 8 nanoseconds of each trajectory. In this analysis, a “contact” defines an interactomic distance (<3 Å) between any pair of atoms occurring in more than 30% of the total simulation frames. Equivalent residues, as defined according to the structural alignment shown in Figure 4C, are reported on the same line with the corresponding energetic contribution (in kJ/mol).
(DOC)

Table S2 **Proportion of variance and cumulative proportion of total variance captured by the first six eigenvectors of the dynamics of free and bound AIRE-PHD1.**
(DOC)

Table S3 **Summary of PHD-H3K4me0 complexes used for MM/PBSA calculations.**
(DOC)

Table S4 **RMSIP values between the eigenvectors obtained from three different time windows (2–5, 2–8 and 2–10 ns) of the free and bound AIRE-PHD1 trajectories (#).**
(DOC)

Table S5 **RMSIP values between the eigenvectors obtained from three different time windows (2–10, 2–30 and 2–50 ns) of the free and bound AIRE-PHD1 trajectories (#).**
(DOC)

References S1 **Supporting Information References.**
(DOC)
