# Supplementary figures and images for: Exploring PHD Fingers and H3K4me0 Interactions with Molecular Dynamics Simulations and Binding Free Energy Calculations: AIRE-PHD1, a Comparative Study
Source: PLoS One. 2012 Oct 15;7(10):e46902. doi: 10.1371/journal.pone.0046902 (PMC3471955; doi:10.1371/journal.pone.0046902)

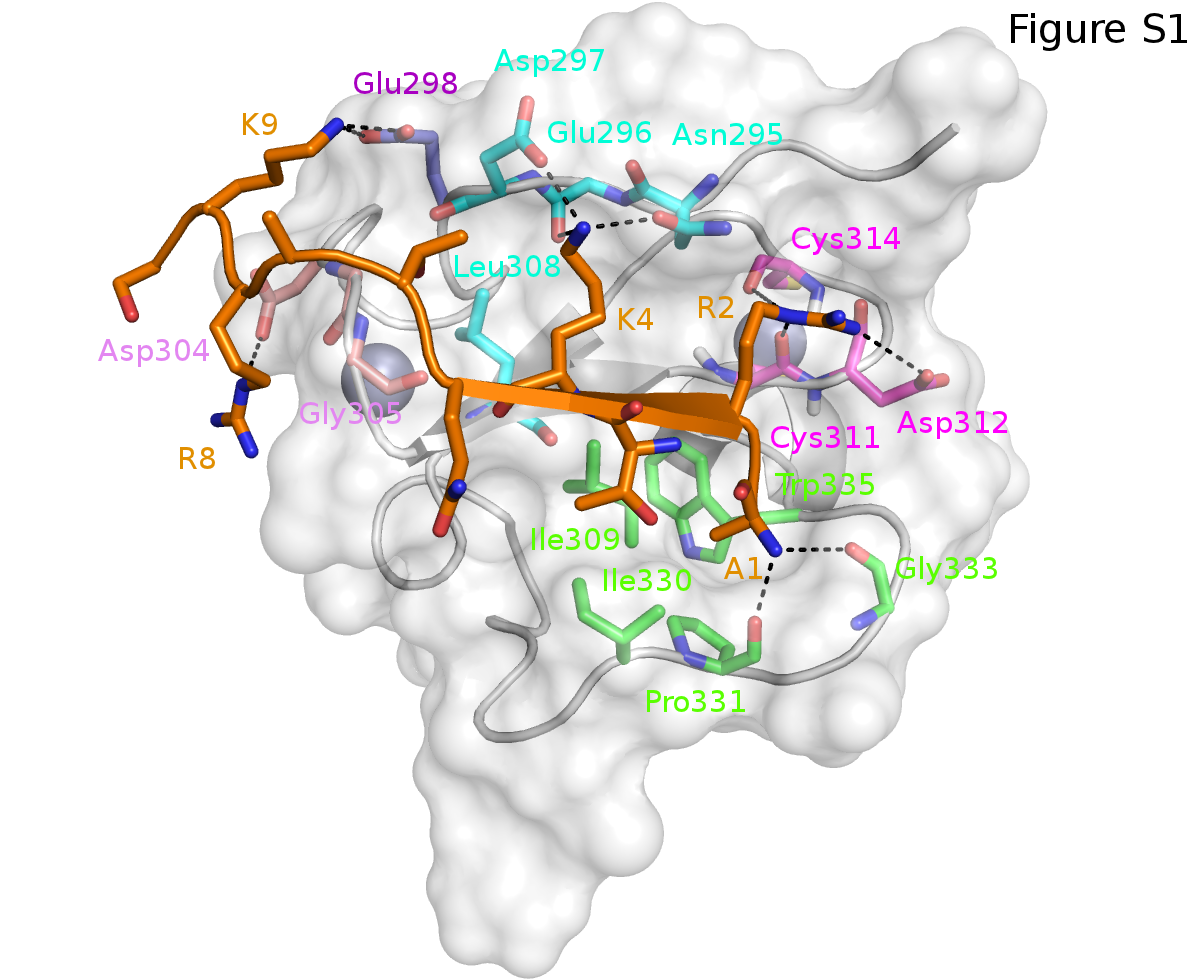

Supplement: Figure S1 — Surface plot of the AIRE-PHD1/H3K4me0 complex. Complex of AIRE-PHD1 (white cartoon and surface) and H3K4me0 (orange cartoon). AIRE-PHD1 residues interacting with H3A1, H3R2, H3K4, H3R8 and H3K9 are shown as green, magenta, cyan, pink and violet sticks, respectively. Dashed lines indicate a selection of the polar contacts of the complex, and Zn2+ ions are represented by grey spheres. (TIFF) [file pone.0046902.s001.tiff]

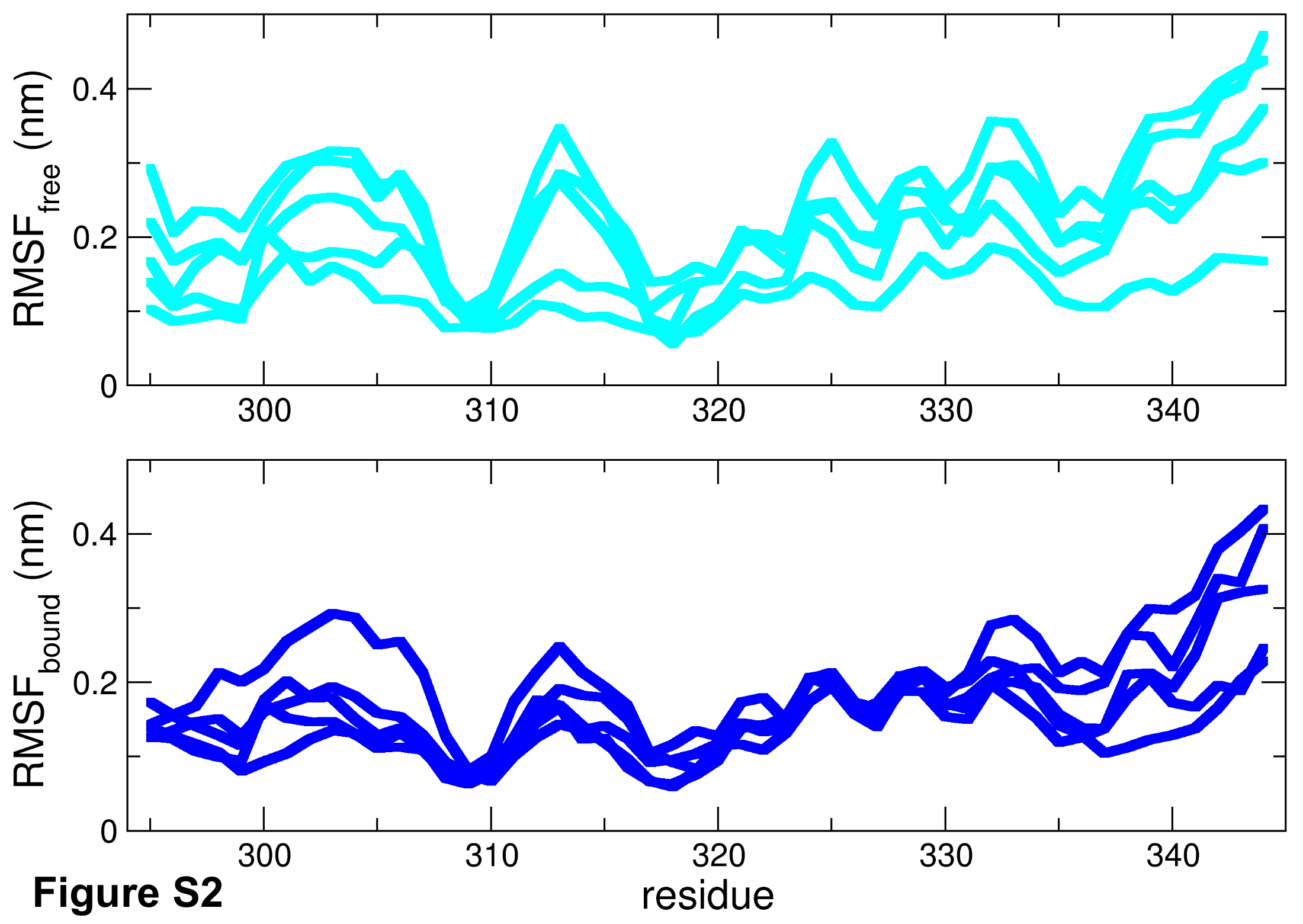

Supplement: Figure S2 — RMSF of Cα atoms from their time-averaged positions for the five replicas of free (cyan) and bound (blue) AIRE-PHD1. (TIFF) [file pone.0046902.s002.tiff]

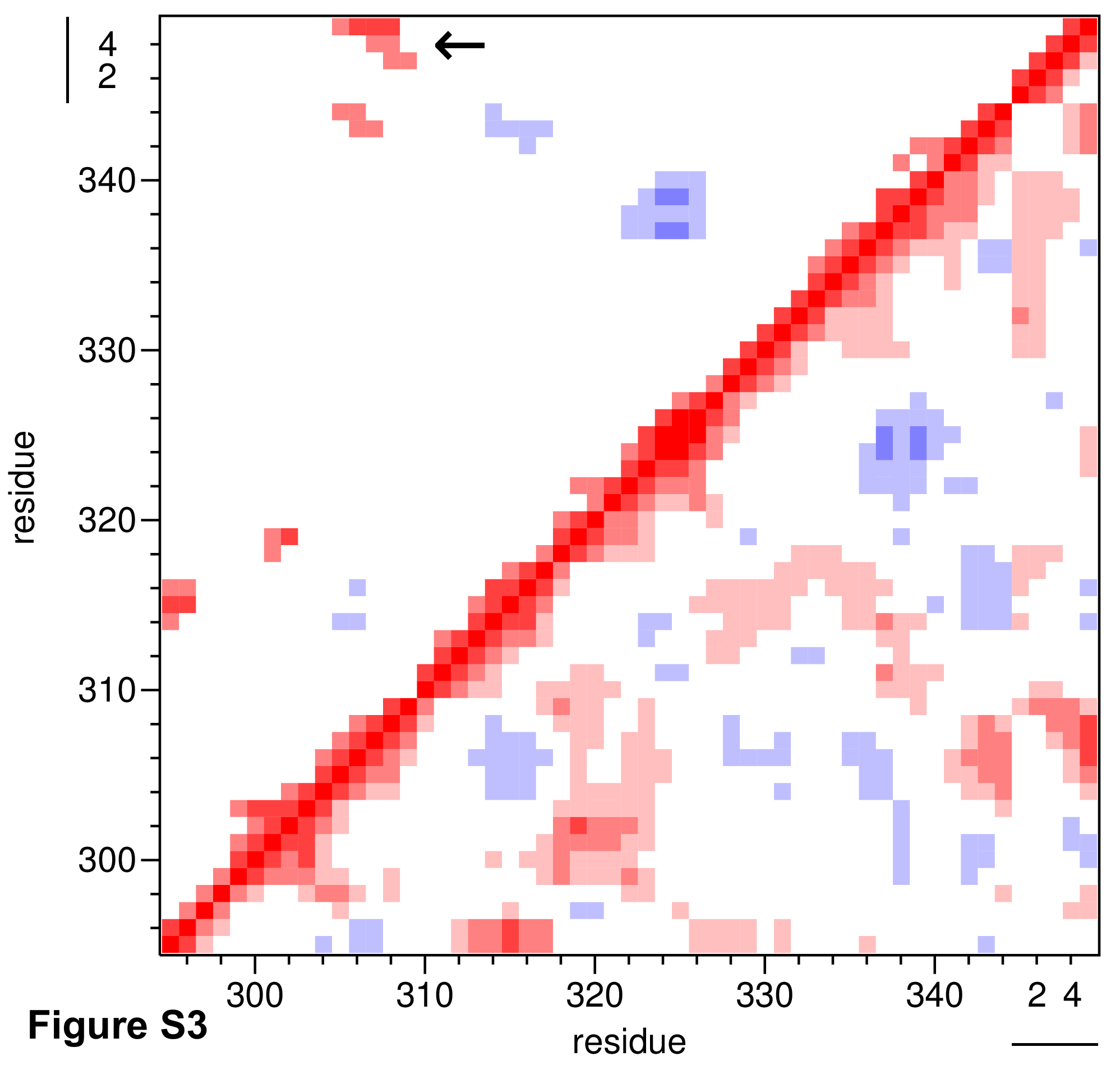

Supplement: Figure S3 — Residue-based (Cα atoms) correlation maps of AIRE-PHD1 (residues 295 to 344) and H3K4me0 (residues 1 to 5, black line). An arrow indicates the correlation between AIRE-PHD1 β1 strand and the additional β strand formed by the histone tail. (TIFF) [file pone.0046902.s003.tiff]

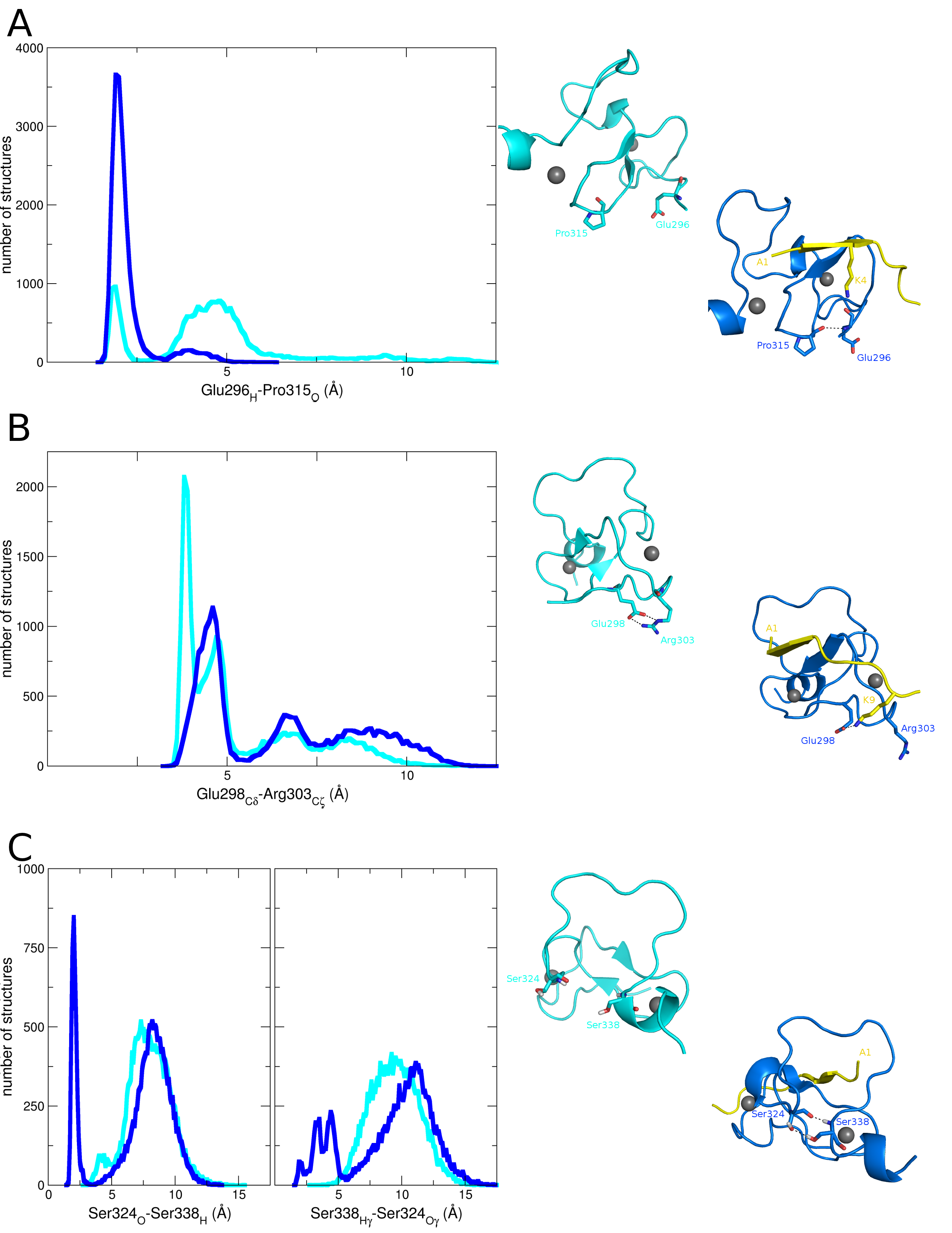

Supplement: Figure S4 — Interactions corresponding to the correlations described by boxes 1, 2 and 3 in Figure 2 . On the left side are shown the distribution of specific distances along the dynamics of free (cyan) and bound (blue) AIRE-PHD1, on the right side are shown two representative structures for free (cyan) and bound (blue) AIRE-PHD1, with grey spheres and yellow cartoon denoting Zn2+ ions and histone tail, respectively. (A) Interaction between the backbone atoms of Glu296 and Pro315, (B) salt-bridge between Glu298 and Arg303 side-chains, (C) Hydrogen bonds between Ser324 and Ser338. (TIFF) [file pone.0046902.s004.tiff]

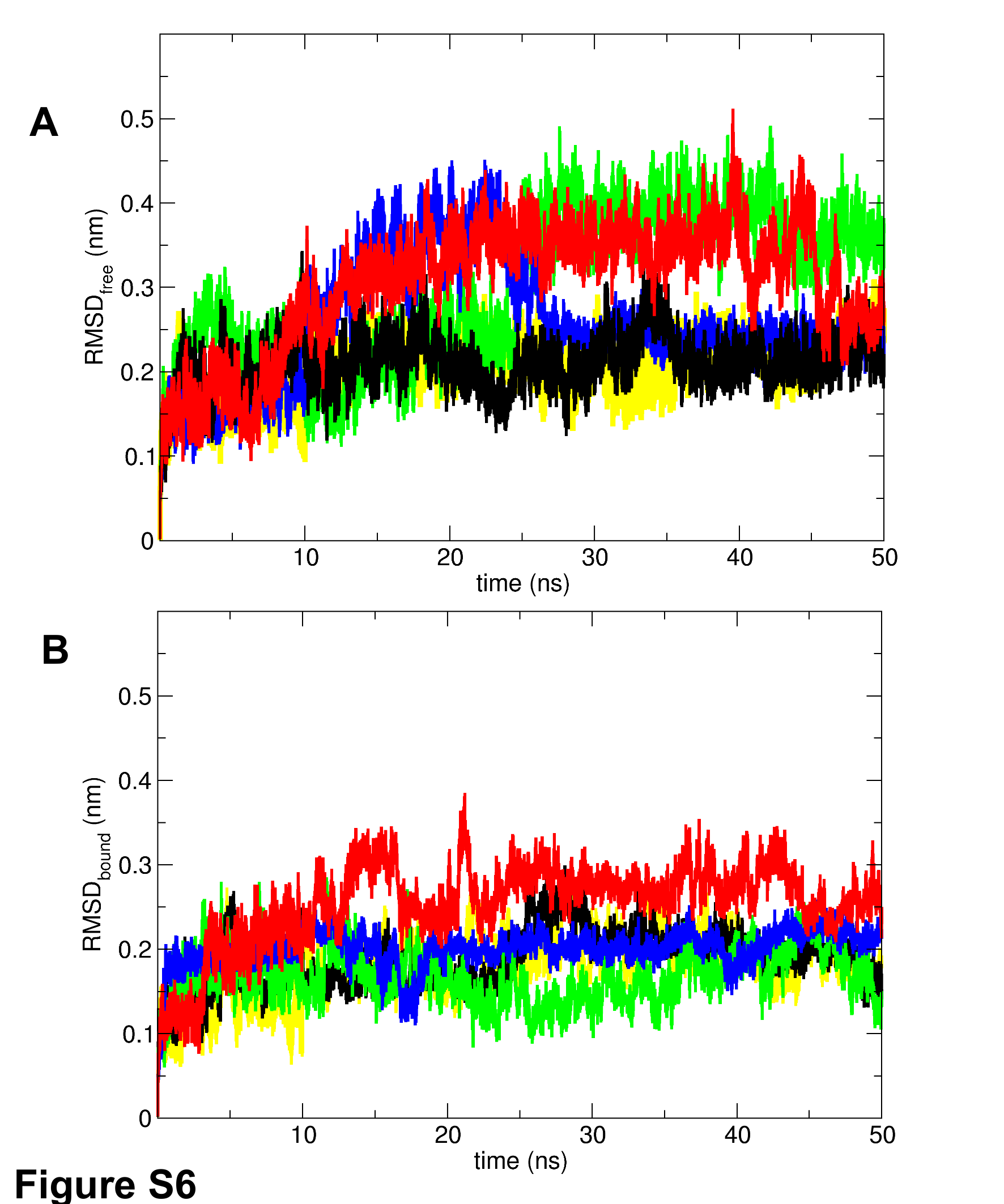

Supplement: Figure S6 — Cα RMSD from (A) free and (B) bound starting AIRE-PHD1 structure, as a function of time in 50 ns trajectories. (TIFF) [file pone.0046902.s006.tiff]
