# Supplementary material for: Exploring PHD Fingers and H3K4me0 Interactions with Molecular Dynamics Simulations and Binding Free Energy Calculations: AIRE-PHD1, a Comparative Study
Source: PLoS One. 2012 Oct 15;7(10):e46902. doi: 10.1371/journal.pone.0046902 (PMC3471955; doi:10.1371/journal.pone.0046902)
Supplement: Table S1 — Interatomic contacts between PHD fingers and H3K4me0 during MD simulations and associated interaction energies. Analyses were performed on the last 8 nanoseconds of each trajectory. In this analysis, a “contact” defines an interactomic distance (<3 Å) between any pair of atoms occurring in more than 30% of the total simulation frames. Equivalent residues, as defined according to the structural alignment shown in Figure 4C, are reported on the same line with the corresponding energetic contribution (in kJ/mol). (DOC) [file pone.0046902.s008.doc]

**Table S1. Interatomic contacts between PHD fingers and H3K4me0 during MD simulations and associated interaction energies**

| **H3 residues** | **AIRE** | **TRIM24** | **CHD4** | **BHC80** | **BRPF2** |
| --- | --- | --- | --- | --- | --- |
| **A1** | Ile309: –2.3  Ile330: –1.1  Pro331: –28.1  Gly333: –46.4  –  Trp335: –4.1 | Leu839: –1.5  –  Pro861: –17.6  Gly863: –43.6  –  Trp865: –4.8 | Leu103: –2.0  Ile124: –1.4  Pro125: –21.2  Gly127: –36.9  –  Trp129: –5.8 | Leu501: –1.9  Ile522: –2.1  Pro523: –28.9  Gly525: –47.7  –  Trp527: –3.7 | Leu43: –1.4  Ile62: –1.3  Pro63: –33.3  Gly65: –41.7  –  Trp67: –3.0 |
| **R2** | Gln293: –10.9  –  Asn295: –0.9  Ile309: –14.7  Cys310: –24.2  Cys311: –23.6  Asp312: –61.9  Cys314: –6.4  – | –  –  –  Leu839: –11.1  Cys840: –21.7  Cys841: –8.8  Glu842: –132.2  –  – | –  –  His89: –9.1  Leu103: –12.3  Cys104: –21.3  Cys105: –8.6  Asp106: –57.9  –  – | –  –  His487: –1.1  Leu501: –13.7  Met502: –24.7  Cys503: –7.1  Asp504: –125.5  –  – | –  –  –  Leu43: –12.1  Phe44: –25.2  –  Asp46: –56.9  –  Gln66: –7.0 |
| **T3** | Gly306: –0.2  –  Ile309: –7.0  –  Ile330: –4.2 | –  –  Leu839: –6.3  –  Phe860: –3.8 | –  Leu102: –18.8  Leu103: –6.9  –  Ile124: –3.2 | –  –  Leu501: –6.9  Met502: –9.6  Ile522: –4.7 | –  –  Leu43: –6.1  –  Ile62: –4.6 |
| **K4** | –  –  Asn295: –55.3  Glu296: –30.2  Asp297: –120.3  Glu307: –10.4  Leu308: –30.8  –  – | –  –  Asn825: –36.7  Glu826: –39.5  Asp827: –108.1  Glu837: –11.0  Leu838: –31.1  Cys840: –5.5  Val847: –1.9 | Asp87: –60.9  –  His89: –18.9  Met90: –10.3  Glu91: –54.2  Glu101: –11.2  Leu102: –31.3  Cys104: –4.8  Ser111: –7.3 | –  –  His487: –22.7  Glu488: –41.5  Asp489: –110.8  Gln499: –14.2  Leu500: –31.3  Met502: –14.4  Val509: –2.1 | Asp23: –128.2  Glu24: –42.2  Ala26: –1.3  –  –  Val41: –13.6  Ile42: –30.3  Phe44: –5.2  – |
| **Q5** | Glu307: –34.7 | Glu837: –41.7 | Glu101: –38.5 | Gln499: –31.9 | Val41: –9.5 |
| **T6** | Asp297: –8.3  Gly305: –13.8  Gly306: –35.7  Leu308: –5.3 | Asp827: –8.3  Gly835: –14.5  Gly836: –33.7  Leu838: –4.4 | Glu91: –9.6  Gly99: –4.8  Gly100: –20.0  Leu102: –3.6 | Asp489: –7.7  Ser497: –10.0  Gly498: –24.6  Leu500: –4.1 | –  Ser39: –13.1  Asn40: –39.0  Ile42: –5.3 |
| **A7** | Gly305: –7.7 | Gly835: –10.2 | Gly99: –9.3 | – | – |
| **R8** | –  –  Asp304: –123.4  Gly305: –9.7  Gly306: –4.2  – | –  –  Asn834: –17.6  Gly835: –10.2  Gly836: –2.6  Glu837: –91.3 | –  –  Asp98: –88.8  Gly99: –12.7  Gly100: –4.1  Glu101: –31.4 | Asp489: –59.8  Phe490: –5.9  –  Ser497: –14.2  –  – | –  –  –  –  Asn38: –18.3  – |
| **K9** | Glu298: –88.6  Arg303: –15.0  Asp304: –30.1  Gly305: –0.9 | –  Gln833: –20.3  Asn834: –26.0  – | –  –  Asp98: –45.8  – | –  –  –  – | Asp25: –57.7  –  –  – |

Analysis were performed on the last 8 nanoseconds of each trajectory. In this analysis, a “contact” defines an interactomic distance (< 3 Å) between any pair of atoms occurring in more than 30% of the total simulation frames. Equivalent residues, as defined according to the structural alignment shown in Figure 4C, are reported on the same line with the corresponding energetic contribution (in kJ/mol).

**Table S2. Proportion of variance and cumulative proportion of total variance captured by the first six eigenvectors of the dynamics of free and bound AIRE-PHD1.**

| **free** | | | **bound** | | |
| --- | --- | --- | --- | --- | --- |
| **eigenvector** | **Proportion of variance (%)** | **Cumulative proportion of variance (%)** | **eigenvector** | **Proportion of variance (%)** | **Cumulative proportion of variance (%)** |
| **1** | 21 | – | **1** | 31 | – |
| **2** | 19 | 40 | **2** | 22 | 53 |
| **3** | 14 | 54 | **3** | 14 | 67 |
| **4** | 7 | 61 | **4** | 7 | 74 |
| **5** | 5 | 66 | **5** | 5 | 79 |
| **6** | 5 | 71 | **6** | 3 | 82 |

Table S3. Summary of PHD-H3K4me0 complexes used for MM/PBSA calculations.

| **complex^1^** | **PDB** | **K_d_ (μM)** | **T (K)** | **ΔG_binding_**  **(kJ/mol)** | **peptide**  **residues** | **technique** | **titration buffer** |
| --- | --- | --- | --- | --- | --- | --- | --- |
| AIRE [1] | 2ke1 | 6.5 | 296 | –29.73 | 10 | ITC | 20 mM phosphate buffer, 150 mM NaCl, 2 mM 2-mercaptoethanol, 50 mM ZnCl_2_ (pH 7.2) [1] |
| TRIM24 [2] | 3o37 | 8.6 | 298 | –28.90 | 10 | ITC | 20 mM Tris, 50 mM NaCl, 2 mM 2-mercaptoethanol (pH 7.5) [2] |
| CHD4 [3] | 2l75 | 18 | 298 | –27.10 | 11 | tryptophan fluorescence | 20 mM sodium phosphate, 150 mM NaCl, 10 mM DTT, 1mM NaN_3_ (pH 7.2) [6] |
| BHC80 [4] | 2puy | 33 | 298 | –25.57 | 10 | ITC | 25 mM Tris-HCl, 50 mM NaCl, 2 mM 2-mercaptoethanol (pH 7.2) [4] |
| BRPF2 [5] | 2l43 | 192 | 293 | –20.85 | 12 | ITC | 50 mM Tris-HCl, 100 mM NaCl (pH 7.5) [5] |

^1^All the structures with the exception of TRIM24 and BHC80 (X-ray) were determined by NMR

**Table S4. RMSIP values between the eigenvectors obtained from three different time windows (2-5, 2-8 and 2-10 ns) of the free and bound AIRE-PHD1 trajectories (#).**

| **Free AIRE-PHD1** | **2 to 5 ns** | | | | | **Bound AIRE-PHD1** | **2 to 5 ns** | | | | |
| --- | --- | --- | --- | --- | --- | --- | --- | --- | --- | --- | --- |
| **MD** | **#1** | **#2** | **#3** | **#4** | **#5** | **MD** | **#1** | **#2** | **#3** | **#4** | **#5** |
| **2 to 8 ns** | 0.93 | 0.93 | 0.92 | 0.91 | 0.93 | **2 to 8 ns** | 0.93 | 0.93 | 0.91 | 0.9 | 0.95 |
| **2 to 10 ns** | 0.92 | 0.91 | 0.91 | 0.88 | 0.91 | **2 to 10 ns** | 0.91 | 0.87 | 0.9 | 0.88 | 0.95 |

**Table S5. RMSIP values between the eigenvectors obtained from three different time windows (2-10, 2-30 and 2-50 ns) of the free and bound AIRE-PHD1 trajectories (#).**

| **Free AIRE-PHD1** | **2 to 10 ns** | | | | | **Bound AIRE-PHD1** | **2 to 10 ns** | | | | |
| --- | --- | --- | --- | --- | --- | --- | --- | --- | --- | --- | --- |
| **MD** | **#1** | **#2** | **#3** | **#4** | **#5** | **MD** | **#1** | **#2** | **#3** | **#4** | **#5** |
| **2 to 30 ns** | 0.89 | 0.86 | 0.88 | 0.89 | 0.93 | **0 to 30 ns** | 0.88 | 0.85 | 0.89 | 0.86 | 0.91 |
| **2 to 50 ns** | 0.88 | 0.80 | 0.85 | 0.86 | 0.86 | **0 to 50 ns** | 0.84 | 0.83 | 0.83 | 0.82 | 0.86 |

**References**

1. Chignola F, Gaetani M, Rebane A, Org T, Mollica L, et al. (2009) The solution structure of the first PHD finger of autoimmune regulator in complex with non-modified histone H3 tail reveals the antagonistic role of H3R2 methylation. Nucleic Acids Res 37: 2951-2961.
2. Tsai WW, Wang Z, Yiu TT, Akdemir KC, Xia W, et al. (2010) TRIM24 links a non-canonical histone signature to breast cancer. Nature 468: 927-932.
3. Mansfield RE, Musselman CA, Kwan AH, Oliver SS, Garske AL, et al. (2011) Plant homeodomain (PHD) fingers of CHD4 are histone H3-binding modules with preference for unmodified H3K4 and methylated H3K9. J Biol Chem 286: 11779-11791.
4. Lan F, Collins RE, De Cegli R, Alpatov R, Horton JR, et al. (2007) Recognition of unmethylated histone H3 lysine 4 links BHC80 to LSD1-mediated gene repression. Nature 448: 718-722.
5. Qin S, Jin L, Zhang J, Liu L, Ji P, et al. (2011) Recognition of unmodified histone H3 by the first PHD finger of bromodomain-PHD finger protein 2 provides insights into the regulation of histone acetyltransferases monocytic leukemic zinc-finger protein (MOZ) and MOZ-related factor (MORF). J Biol Chem 286: 36944-36955.
6 . Musselman CA, Mansfield RE, Garske AL, Davrazou F, Kwan AH, et al. (2009) Binding of the CHD4 PHD2 finger to histone H3 is modulated by covalent modifications. Biochem J. 423(2):179-87
