# Supplementary material for: Exploring PHD Fingers and H3K4me0 Interactions with Molecular Dynamics Simulations and Binding Free Energy Calculations: AIRE-PHD1, a Comparative Study
Source: PLoS One. 2012 Oct 15;7(10):e46902. doi: 10.1371/journal.pone.0046902 (PMC3471955; doi:10.1371/journal.pone.0046902)
Supplement: Table S2 — Proportion of variance and cumulative proportion of total variance captured by the first six eigenvectors of the dynamics of free and bound AIRE-PHD1. (DOC) [file pone.0046902.s009.doc]

**Table S2. Proportion of variance and cumulative proportion of total variance captured by the first six eigenvectors of the dynamics of free and bound AIRE-PHD1.**

| **free** | | | **bound** | | |
| --- | --- | --- | --- | --- | --- |
| **eigenvector** | **Proportion of variance (%)** | **Cumulative proportion of variance (%)** | **eigenvector** | **Proportion of variance (%)** | **Cumulative proportion of variance (%)** |
| **1** | 21 | – | **1** | 31 | – |
| **2** | 19 | 40 | **2** | 22 | 53 |
| **3** | 14 | 54 | **3** | 14 | 67 |
| **4** | 7 | 61 | **4** | 7 | 74 |
| **5** | 5 | 66 | **5** | 5 | 79 |
| **6** | 5 | 71 | **6** | 3 | 82 |
