# Supplementary material for: Exploring PHD Fingers and H3K4me0 Interactions with Molecular Dynamics Simulations and Binding Free Energy Calculations: AIRE-PHD1, a Comparative Study
Source: PLoS One. 2012 Oct 15;7(10):e46902. doi: 10.1371/journal.pone.0046902 (PMC3471955; doi:10.1371/journal.pone.0046902)
Supplement: Table S3 — Summary of PHD-H3K4me0 complexes used for MM/PBSA calculations. (DOC) [file pone.0046902.s010.doc]

Table S3. Summary of PHD-H3K4me0 complexes used for MM/PBSA calculations.

| **complex^1^** | **PDB** | **K_d_ (μM)** | **T (K)** | **ΔG_binding_**  **(kJ/mol)** | **peptide**  **residues** | **technique** | **titration buffer** |
| --- | --- | --- | --- | --- | --- | --- | --- |
| AIRE [1] | 2ke1 | 6.5 | 296 | –29.73 | 10 | ITC | 20 mM phosphate buffer, 150 mM NaCl, 2 mM 2-mercaptoethanol, 50 mM ZnCl_2_ (pH 7.2) [1] |
| TRIM24 [2] | 3o37 | 8.6 | 298 | –28.90 | 10 | ITC | 20 mM Tris, 50 mM NaCl, 2 mM 2-mercaptoethanol (pH 7.5) [2] |
| CHD4 [3] | 2l75 | 18 | 298 | –27.10 | 11 | tryptophan fluorescence | 20 mM sodium phosphate, 150 mM NaCl, 10 mM DTT, 1mM NaN_3_ (pH 7.2) [6] |
| BHC80 [4] | 2puy | 33 | 298 | –25.57 | 10 | ITC | 25 mM Tris-HCl, 50 mM NaCl, 2 mM 2-mercaptoethanol (pH 7.2) [4] |
| BRPF2 [5] | 2l43 | 192 | 293 | –20.85 | 12 | ITC | 50 mM Tris-HCl, 100 mM NaCl (pH 7.5) [5] |

^1^All the structures with the exception of TRIM24 and BHC80 (X-ray) were determined by NMR
