# Supplementary material for: Exploring PHD Fingers and H3K4me0 Interactions with Molecular Dynamics Simulations and Binding Free Energy Calculations: AIRE-PHD1, a Comparative Study
Source: PLoS One. 2012 Oct 15;7(10):e46902. doi: 10.1371/journal.pone.0046902 (PMC3471955; doi:10.1371/journal.pone.0046902)
Supplement: Table S5 — RMSIP values between the eigenvectors obtained from three different time windows (2–10, 2–30 and 2–50 ns) of the free and bound AIRE-PHD1 trajectories (#). (DOC) [file pone.0046902.s012.doc]

**Table S5. RMSIP values between the eigenvectors obtained from three different time windows (2-10, 2-30 and 2-50 ns) of the free and bound AIRE-PHD1 trajectories (#).**

| **Free AIRE-PHD1** | **2 to 10 ns** | | | | | **Bound AIRE-PHD1** | **2 to 10 ns** | | | | |
| --- | --- | --- | --- | --- | --- | --- | --- | --- | --- | --- | --- |
| **MD** | **#1** | **#2** | **#3** | **#4** | **#5** | **MD** | **#1** | **#2** | **#3** | **#4** | **#5** |
| **2 to 30 ns** | 0.89 | 0.86 | 0.88 | 0.89 | 0.93 | **0 to 30 ns** | 0.88 | 0.85 | 0.89 | 0.86 | 0.91 |
| **2 to 50 ns** | 0.88 | 0.80 | 0.85 | 0.86 | 0.86 | **0 to 50 ns** | 0.84 | 0.83 | 0.83 | 0.82 | 0.86 |
