# Supplementary material for: Exploring PHD Fingers and H3K4me0 Interactions with Molecular Dynamics Simulations and Binding Free Energy Calculations: AIRE-PHD1, a Comparative Study
Source: PLoS One. 2012 Oct 15;7(10):e46902. doi: 10.1371/journal.pone.0046902 (PMC3471955; doi:10.1371/journal.pone.0046902)
Supplement: References S1 — Supporting Information References. (DOC) [file pone.0046902.s013.doc]

**Supplementary References**

1. Chignola F, Gaetani M, Rebane A, Org T, Mollica L, et al. (2009) The solution structure of the first PHD finger of autoimmune regulator in complex with non-modified histone H3 tail reveals the antagonistic role of H3R2 methylation. Nucleic Acids Res 37: 2951-2961.
2. Tsai WW, Wang Z, Yiu TT, Akdemir KC, Xia W, et al. (2010) TRIM24 links a non-canonical histone signature to breast cancer. Nature 468: 927-932.
3. Mansfield RE, Musselman CA, Kwan AH, Oliver SS, Garske AL, et al. (2011) Plant homeodomain (PHD) fingers of CHD4 are histone H3-binding modules with preference for unmodified H3K4 and methylated H3K9. J Biol Chem 286: 11779-11791.
4. Lan F, Collins RE, De Cegli R, Alpatov R, Horton JR, et al. (2007) Recognition of unmethylated histone H3 lysine 4 links BHC80 to LSD1-mediated gene repression. Nature 448: 718-722.
5. Qin S, Jin L, Zhang J, Liu L, Ji P, et al. (2011) Recognition of unmodified histone H3 by the first PHD finger of bromodomain-PHD finger protein 2 provides insights into the regulation of histone acetyltransferases monocytic leukemic zinc-finger protein (MOZ) and MOZ-related factor (MORF). J Biol Chem 286: 36944-36955.
6 .Musselman CA, Mansfield RE, Garske AL, Davrazou F, Kwan AH, et al. (2009) Binding of the CHD4 PHD2 finger to histone H3 is modulated by covalent modifications. Biochem J. 423(2):179-87
